# Use of Action Research in Nursing Education

**DOI:** 10.1155/2016/8749167

**Published:** 2016-12-18

**Authors:** Susan D. Moch, R. Todd Vandenbark, Shelley-Rae Pehler, Angela Stombaugh

**Affiliations:** ^1^Department of Nursing, College of Nursing and Health Sciences, University of Wisconsin-Eau Claire, 105 Garfield Avenue, P.O. Box 4004, Eau Claire, WI 54702-4004, USA; ^2^Vogel Library, Wartburg College, No. 225, 100 Wartburg Blvd, P.O. Box 1003, Waverly, IA 50677, USA; ^3^Department of Nursing, College of Nursing and Health Sciences, No. 219 Nursing Building, University of Wisconsin-Eau Claire, 105 Garfield Ave, P.O. Box 4004, Eau Claire, WI 54702, USA; ^4^Center for Excellence in Teaching and Learning, University of WI-Eau Claire, Eau Claire, WI, USA

## Abstract

*Purpose.* The purpose of this article is to describe action research in nursing education and to propose a definition of action research for providing guidelines for research proposals and criteria for assessing potential publications for nursing higher education.* Methods.* The first part of this project involved a search of the literature on action research in nursing higher education from 1994 to 2013. Searches were conducted in the CINAHL and MEDLINE databases. Applying the criteria identified, 80 publications were reviewed. The second part of the project involved a literature review of action research methodology from several disciplines to assist in assessing articles in this review.* Results.* This article summarizes the nursing higher education literature reviewed and provides processes and content related to four topic areas in nursing higher education. The descriptions assist researchers in learning more about the complexity of both the action research process and the varied outcomes. The literature review of action research in many disciplines along with the review of action research in higher education provided a framework for developing a nursing-education-centric definition of action research.* Conclusions.* Although guidelines for developing action research and criteria for publication are suggested, continued development of methods for synthesizing action research is recommended.

## 1. Introduction

Despite the call for knowledge development in nursing education [1–3] and concerns about the lack of dissemination of nursing education knowledge [4, 5], limited research to guide nursing education is available. The use of action research in knowledge development and in assessing nursing knowledge for publications could increase nursing knowledge. At its core, action research methodology involves a systematic research process and thoughtful reflection on the process for making a change. The purpose of this article is to describe action research in nursing education and to propose a definition of action research that provides both guidelines for action research proposals and criteria for potential action research publications in nursing higher education.

Although publications on action research are available in nursing higher education in some countries, the use of action research is much more prevalent in related disciplines. Use of action research is evident in addressing health disparities [6, 7], leadership/organization development [8–11], and nursing practice in general [12]. Action research is also widely used within all levels of education. At the K-12 level, research is often discussed as personal or collaborative reflection to delineate educational outcomes [13–15]. According to some authors, action research in higher education makes the research more applicable to the real world through combining research and practice [16, 17]. In 2005, Herr and Anderson [18] described how action research proposals and dissertations are evaluated. However, acceptance of action research in higher education has been difficult because of the historical focus on empirical research and behavioral outcomes [19, 20] and because of misconceptions about action research itself [21].

Due to the increasing use of action research in many disciplines and the need for outcome research in nursing higher education, a review of action research in nursing higher education literature was undertaken to describe action research in nursing education. A definition of action research that provides both guidelines for developing action research proposals and criteria for assessing potential action research publications for nursing higher education was developed. And much like action research methodology, the process used in this review study was iterative, in that, after a review was completed, it became apparent that a clear, consistent, and actionable definition was needed. Using existing definitions and ideas from several disciplines, the authors propose a new definition of action research. For this project, the definition proposed was used in assessing the body of literature on nursing higher education research.

## 2. Methods

### 2.1. Literature Review Process of Action Research in Nursing

The first part of this project involved a search of the literature in nursing higher education related to action research. Searches were conducted in the CINAHL and MEDLINE databases using the search string* “action research” AND nurs* ^*∗*^ *AND education*, including all articles from 1994 to 2013. Initially, 386 articles were retrieved. They were initially evaluated based on review of each title and abstract, eliminating those articles that were dissertations, conference abstracts, or articles pertaining to nursing practice. Also eliminated were items not directly related to nursing education, such as postdegree continuing education, as well as those that did not offer a description of a systematic research processes. Since few of the articles before 2003 described the research process and met the criteria identified, a decision was made to eliminate those published before 2003, leaving 80 publications.

While completing the initial review, the research team found that not having a consistent definition for action research hampered their efforts for determining which articles should be included in the review. Based on the initial review of articles, a working definition for action research was developed (described in detail below). The remaining 80 citations were then reviewed in detail, with an additional 41 being articles eliminated because they did not meet the criteria of the author definition as identified from a second general literature review on action research definitions. The final inclusion criteria for the higher education research articles were as follows:

- Published between 2003 and 2013
- Related to higher education in nursing
- Indicated which action research methodology was used
- Included a clear description of the research methodology
- Stated which data collection processes were employed
- Analyzed findings and/or process of the research

**Figure 1:**
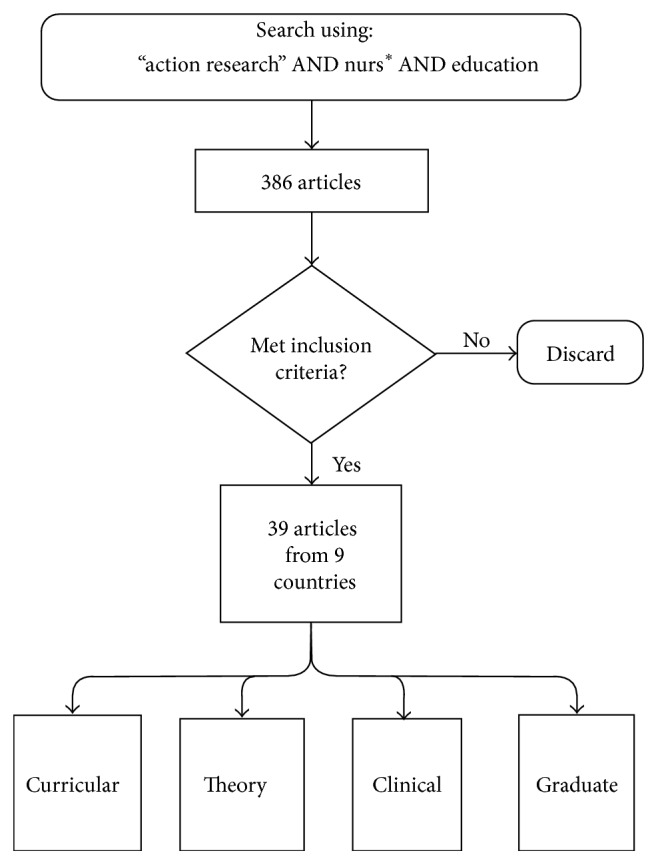


This process is diagrammed in Figure 1.

## 3. Results and Discussion

### 3.1. Building a Definition of Action Research

The many variations in approaches to action research became apparent in the nursing education literature review. Reviewing the nursing articles initially included looking at all research articles that included action research in nursing higher education. However, as stated earlier, upon review of the higher education articles, many variations of “action research” were found. Therefore, a review of definitions and key concepts of action research was completed to develop criteria for inclusion of articles for the review. The following paragraphs include both definitions and key concepts of action research from a variety of disciplines.

Action research methods are based on many broad philosophical and theoretical traditions such as Freire [22], Lewin [23], and Schön [11]. The various traditions and the manner in which action research is employed in the disciplines provide great diversity in action research. In addition, definitions of action research within disciplines can often emphasize different components. Discussion of these traditions and/or definitions within the research report can provide important information for framing the research and is suggested. For instance, within the body of organizational development literature, action research is “a term for describing a spectrum of activities that focus on research, planning, theorizing, learning, and development” which includes a “process of research and learning through the researcher's long-term relationship with a problem” [24, p. 4]. Another important reference to action research in education clarifies that both* action* and* research* are involved in the term so that systematic data collection processes must be in place for the research component [25]. The “action” in the research can involve evaluation of a process being used or demonstration of a change over time, both of which require clear data collection processes. Action research, often used in educational research [26], is promoted as a way to integrate teaching and learning [27]. Kemmis [28] promotes the importance of both knowledge and change as results through action research. From a social sciences perspective, Reason and Bradbury [29] describe action research as a participatory process for developing practical knowledge for solutions to issues of importance. In a later article, Bradbury Huang [21] emphasized the importance of the integration of practice and research or the merging of “understand and act” (pg. 93) and promote “actionability” (pg. 103) as important to the research process. In the same article, Bradbury Huang also shared “suggestions” for publishing articles in the* Action Research* journal.

Within nursing, action research definitions have been proposed and used in the organization of reported research. Margaret Newman, an early nurse theorist, proposed an action research definition in* Theory Development in Nursing* [30]. According to Newman, action research involves “the collaboration of the researcher in the real-world situation of the client system with the purposes of improving the situation, developing the competencies of the system, and generating new knowledge” (p. 71). Although useful for a general understanding of the action research process, this definition does not provide enough direction for nurse educators in developing research and publishing outcomes. Winter and Munn-Giddings [31] describe action research as a “single activity which is simultaneously a form of inquiry and a form of practical action” (p. 5) and “involves people in a process of change, which is based in professional, organizational or community action” (p. 5). Four types of research foci for action research were proposed by Hart and Bond [32] and used to organize research in a nursing-related evidence-based practice article [12]. The four types, based on the theoretical underpinnings of action research, included the following: experimental, organizational, professionalizing, and empowering.

In a systematic literature review of action research published in the* Journal of Research in Nursing*, Munn-Giddings and colleagues found 24 different names attributed to the action research method [33]. Terms often associated with action research in nursing include community-based action research, participatory action research, appreciative inquiry, cooperative inquiry, praxis, process knowledge, reflective practice, and pattern recognition. Although definitions for some of these research processes exist, the definitions do not provide enough direction for nursing education research and publication.

### 3.2. Action Research Definition

*Action research methodology is a systematic research process that can be articulated by the researcher, involving data collection and analysis as well as reflection and discussion with coresearchers or others for the purpose of making change in a situation over time*.

This proposed definition addresses a number of shortcomings in the existing literature, both in and out of nursing education. It can provide researchers with a common starting point for conducting higher education research, where, because of the stage of knowledge development, the participation of collaborators in change, or for other reasons, using more traditional research through involving control groups, large numbers of subjects, or multiple sites may not be appropriate.

The definition calls for a* systematic research process that can be articulated by the researcher*, which assists in developing and publishing research. Adding the component of* that can be articulated by the researcher* is important because action research can include great variability in process, but the need for describing how the research was done is important to research development and dissemination in nursing education.* Involving data collection and analysis* is essential for identifying research outcomes as called for in nursing.

The components of* reflection and discussion with coresearchers or others* include the need to demonstrate reflection on the research in order to consider important factors that may affect the outcomes. The discussion component also includes the possibility of collaboration with practice partners to affect change or the implementation of the research. Including collaborations with coresearchers such as students or community partners in the development and process of the research project is also possible. At times, reflection includes representatives of those directly affected by any proposed changes, such as in participatory action research or community-based collaboration. While this methodology directly involves the researchers in an iterative and reflective research process, varying degrees of participation can be employed [34]. Reflection and discussion with coresearchers may be less relevant in some nursing education contexts.

*Making a change in a situation over time* implies that such research is primarily concerned with outcomes such as change leading to improvement with quality data and analysis resulting in dissemination of knowledge. Making change is important in action research and is implied in its name, including and involving both action (change) and research. This often involves engagement with a project for several years, such as continual improvement in learning outcomes which contributes to change over time. It is collaborative in nature, with the researcher(s) playing the part of active participant or actor in the research. And the power of this role can be used to promote change and create new knowledge [35].

As described previously, the author definition provided a general framework for exclusion of articles within the action research in higher education literature review. The final 39 articles selected included a systematic research process, and many, but not all, incorporated* reflection and discussion with coresearchers or others for the purpose of making change in a situation over time*. However, since the components of the action research definition emerged from both the review of action research definitions and the nursing education action research articles, a decision was made to retain* reflection and discussion with coresearchers or others* and* making change in a situation over time* in the definition.

### 3.3. Applying the Definition Structure

The following is a summary of the action research articles found in nursing higher education. Summarizing action research is challenging because all elements of the action research process and its outcomes (such as all the components of the definition) are important for understanding the research. For instance,* a systematic research process that can be articulated by the researcher* may be different for each study due to the use of different yet valid processes. Often the description of the research is detailed and includes how the collaborators were involved or how data was collected through meetings, discussions, or surveys. In addition, challenges arise in summarizing this particular literature research because the topic of  “action research in nursing higher education” is very broad. No narrowing of the research focus was used in this study, so many different topics, processes, and collaborations are included. Thus the articles are organized according to four general topic areas in nursing higher education with the purpose of providing an overview of the subject matter and the types of action research available. The author intent is to encourage understanding about the possibilities that exist in conducting action research in regard to content focus as well as in variety of methodology. A summary table of some information is included (see Table 1), but the central idea or topic of the articles within each group is also presented in narrative form through summaries to assist readers and potential future researchers considering an action research approach. To facilitate greater understanding of this methodology, the research process from one article is highlighted at the end of each summary to demonstrate action research process within each topic area. Within each article example, action research issues such as the research tradition, dilemmas in conducting the research, and identified “action” for the research are also shared if identified in the research report.

The general topic areas are as follows:

- Theory: research conducted in a nonclinical, in-classroom setting, often with a focus on knowledge sharing
- Clinical: instruction-related research with an experiential component
- Curricular: research applied to nursing curriculum at the department or school/college level
- Graduate: focus on research with students in nursing graduate programs.

### 3.4. Theory

The use of action research methodology to improve or enrich student learning in nursing education ranged from the individual instructor to the class as a whole and from lecture content to facilitating professional development. Research within the classroom involved student feedback that shifted the focus of lectures from the instructor to the students [36]; improved the structure and effectiveness of cooperative learning activities [37]; refocused course content from family health policy to clinical ethics [38]; incorporated artistic aspects of the humanities into two graduate-level nursing classes [39]; and helped deepen nursing students' understanding of the challenges of living in poverty [40]. Also, a participatory action research approach helped administrators better understand the needs of clinical facilitators who supervise student nurses which led to increased feedback and mentoring among facilitators [41]. The diversity in approaches, settings, and areas of focus demonstrate how action research can bring real and immediate improvements to nursing education courses.

As an example of how one article discusses the action research process, Smith-Stoner and Molle [37] sought to develop a systematic way of implementing cooperative learning in the nursing classroom using action research to evaluate their efforts. They wanted to determine if cooperative learning could improve learning outcomes. The authors cite several theoretical and action research definition sources and note that classroom action research was used. Their research progressed through four cycles of action followed by reflection, with student feedback serving as the data collected each step of the way. Meeting every two weeks, faculty reflected on their successes, challenges, and student reactions to the variation in instruction format. Limited dilemmas were reported and the action portion of the research involved changes in classes that was articulated and instituted. The reflection and discussion among the “actors” or coresearchers also encouraged learning about teaching. Implications for using cooperative learning with nursing students are identified.

### 3.5. Clinical

For the articles related to the clinical experience, the purpose of the action research design varied greatly. Research that included patients or the community allowed students to partner with parents who have children with disabilities [42] and to help a community prepare for a disaster [43]. Other articles involved working to improve communication between students and nurses in the clinical setting [44]; developing a new teaching strategy to improve therapeutic communication in nursing students [45]; designing a new clinical role to improve the theory/practice gap [46]; exposing students to working in long-term care [47]; developing interprofessional collaboration during simulation-based learning [48]; and applying new strategies for student evaluation. Some student evaluation strategies included a portfolio approach [49] and patient involvement in student evaluation [50]. Such a variety of situations and problems addressed by action research in the reviewed literature demonstrates how action research was used to increase nursing outcome knowledge in clinical aspects of nursing education.

Research on efforts to attract nursing students to aged care [47] provides an example of applying action research in a clinical setting. In order to overcome nursing students' perception of caring for the elderly as unattractive at best, researchers and nurse preceptors collaborated to create a more welcoming and supportive orientation for student nurses. Applying Kemmis' [51] definition of action research, Robinson and colleagues facilitated critical self-reflections and critique among the preceptors in order to “develop and implement strategies to address problem issues” (p. 356). The research processes are clearly described and demonstrate change over time. Data is collected and analyzed at each stage and presented in table form as quotes, combined with a set of recommendations. Dilemmas in conducting the research are not described, but the actions on the part of the researchers and nurse preceptors demonstrated a difference in student perceptions of aged care.

### 3.6. Curricular

Twenty-three articles in this review focused on the curricular aspects of nursing education. While resulting changes varied greatly, five general themes emerged in the use of an action research approach. First, concepts infused throughout the curriculum were often explored, including professional identity [52], caring [53], and cultural safety [54]. A second theme was action research that required collaboration and partnerships among hospitals and universities to advance the curricula [55–60]. Third, action research was used to initiate curricula change related to degree requirements [61, 62] and assessment [49, 63, 64]. Fourth, a number of articles focused on making innovative curricular changes to address student or faculty concerns [65–70]. Action research was also used to focus on student selection [71] and retention [72, 73] as well as encouraging student involvement in faculty research [74]. The large volume of articles in the literature suggests that the method is fluid enough to work with complicated problems that stretch beyond one course.

One research example that demonstrates change over time described a several year action research process that encouraged dialogue and identified outcomes related to involving practice partners in the educational process [56]. The study focused on perceived needs of practice partners to continue collaboration within the university despite no payment for clinical teaching and other services. Participatory action research was cited as the tradition or definition used in the research process. The many political and contextual variables related to payment and volunteer involvement by practitioners were identified through the process. The identification of these variables involved making frequent decisions about how to include the information from the many data sources such as meeting notes, discussions, interviews, reflective diaries and formal reports. The researchers also discussed continued attention to the “participatory” nature of the action research process used. The action aspect of the study identified the complex dialogue, the participatory process involved, and the possible strategies for university leaders to adopt in regard to continued collaboration with service partners.

### 3.7. Graduate

A majority of the curricular articles reviewed focus on undergraduate and certificate-level programs. The investigations into changes at the graduate level involved evaluation and development of graduate curricula with practice partners, students, and educators. Articles that focused on advanced-practice clinical or community health included using portfolios to prompt student self-evaluation of learning [63], developing a framework for organizational partnerships in midwifery education [55] and sharing qualitative data from meetings and focus groups in evaluating unpaid clinical supervision in a university setting [56]. Other graduate-related research included creating an alternative yet sustainable model of online learning through flexible curriculum design [75], critically examining the implementation of a specialist in nursing education postgraduate degree offered by 5 universities over 2.5 years [62]; integrating the humanities into graduate-level nursing education [39]; involving advanced-practice nursing students in faculty research [74]; incorporating research, evaluation, and reflection into daily teaching practice [38]; facilitating effective online student interactions [66]; mentoring young, emerging nursing leaders as a part of succession-planning [69]. These publications offer concrete examples of how an action research approach could enhance and enrich the graduate education experience.

A community health example of action research in nursing education outlines a very significant but difficult area to study using more traditional research approaches [67]. The authors describe a collaborative approach to developing course materials with community teaching partners and students while incorporating collaborative decision making and the various contexts involved with student experiences. The authors describe the research tradition of cooperative inquiry used and articulate the collaborative process between faculty, providers and students. Outcomes for the project include specific modules such as “Specialist Nursing in the Home” that was developed through dialogue between students and clinical teaching partners to assist students in learning about making clinical decisions together while employing knowledge of the home and neighborhood context. The outcomes of this project provides impetus for action for others interested in a similar topic and also provides examples of course modules. In action research methodology, both the process of the research and the product of the research provide knowledge and contribute to the “actionability” of the project. The process of this research provided many dilemmas, such as choosing the research tradition, concern about identifying outcomes, and time necessary for self-reflection among the researchers. Initially the group sought to include students and community practice partners in the self-reflective process, but due to the outside demands of time for these groups, the self-reflection component of the project included only nurse lecturers.

## 4. Conclusions

This review provides an overview of the use of action research in nursing higher education literature. Because a previous review of practice articles included no definition of action research for this field [33], a clear and actionable definition was conceived by the authors of this paper. The definition was based on both a review of action research definitions and a review of action research articles in nursing education. Many components of the definition were then used to critically evaluate the body of literature on action research in higher education. Many articles found in this search had a limited or nonexistent description of action research methodology or lacked a systematic data collection process and were therefore excluded.

Even though Newman [30] advocated early on for use of action research in nursing due to the direct connection to practice, limited use is evident in nursing education. Clear and reproducible examples guided by an action research definition are needed to spotlight the research potential in the types of instruction and critical evaluation projects nurse educators complete. And while many of the articles provide evidence of outcomes for nursing education, summarizing the existing literature was made more difficult by the myriad and diverse ways action research has been applied to nursing higher education. The following recommendations were formulated to facilitate the development of more outcome knowledge for nursing higher education:

1. Use the clear and actionable definition provided in this review for applying criteria for research development.
2. Use the definition to provide publishers and reviewers criteria for reviewing potential action research publications.
3. Develop and refine a method for efficiently and effectively sharing action research summaries for use in nursing education.

New knowledge to improve nursing higher education is needed and use of action research can help fill this gap. Through action research, variables such as context, collaboration with others, and change over time can be incorporated into the research. Enhancement of nursing education should include further development of effective knowledge transfer to clinical practice, process and outcome for interdisciplinary learning, best practices for learning about teaching for nursing professors, and skills for enabling use of research evidence for future clinicians. Some examples of possible action research projects that include current trends in healthcare include the following:

- Nursing faculty and interdisciplinary community healthcare teams collaborating to identify student roles in health promotion for obesity
- Within dedicated units in hospitals, nursing faculty and students, together with hospital staff develop clinical experiences across time that improve student learning while giving back to the unit
- Community partners joining with nursing students and faculty to promote the needs of young children in lieu of an acute care pediatric experience.

## 5. Summary

A review of publications in nursing education literature on this topic resulted in narrative reporting in four topic areas: theory, clinical, curricular, and graduate. A nursing-education-centric definition of action research was created based on the nursing literature review and review of action research in many disciplines and then used to evaluate nursing literature. The definition can also be used for assessing potential action research publications. Many of the articles initially reviewed lacked either a clear methodology or systematic data collection. This article summarizes the literature reviewed and provides topics, processes, and outcomes related to several areas in nursing higher education. The descriptions and discussions of the four examples from each topic area can assist researchers in learning more about the complexity of both the action research process and the varied outcomes. Although guidelines for developing action research and criteria for publication are suggested through the definition, the continuing development of methods for synthesizing the research is also essential for knowledge development.

## Figures and Tables

**Table 1:** Overview of review articles' focus, participants/researchers, and data collection tools.

Area of nursing education	Number of articles	Participants/researchers	Data tools
Theory	7	Students, instructors, supervisors	Surveys, semistructured feedback sessions and interviews, focus groups, reflective journaling
Clinical	12	Patients, community or health agency, nurses, nursing students	Surveys, questionnaires, qualitative data collection
Curriculum	23	Faculty, students, nurses, educators, physicians, preceptors, administration, community members, nurse midwives, managers, government professionals, industry, community health providers	Interviews, surveys, field notes, diaries, team meetings, end semester reflection meetings, focus groups, questionnaires, workshop, reflective journals, curriculum documents, teacher notes, patchwork text, exams, group reports, class participation, observations
Graduate	13	Students, instructors, supervisors (clinical partners), community health staff, users of services/carers	Meeting notes, description of process, self-reflective process, surveys, focus groups, interviews, discussion, reflective writing
